# Simulation-based training of surgical skills

**DOI:** 10.1007/s40037-016-0251-y

**Published:** 2016-01-21

**Authors:** L. Konge, L. Lonn

**Affiliations:** Copenhagen Academy for Medical Education and Simulation, The Capital Region of Denmark, Rigshospitalet, and University of Copenhagen, Copenhagen, Denmark; Departments of Radiology and Vascular Surgery, Rigshospitalet, and University of Copenhagen, Copenhagen, Denmark

In this edition of Perspectives on Medical Education, Nesbitt et al. explore the role of simulation in the development of surgical skills [1]. The author’s main purpose of interest was the literature concerning endovascular simulation. The acquisition of motor skills is, however, universal and therefore their findings can be combined with research from other surgical specialities and in the end generalized into evidence-based guidelines. An extensive review from 2011 compared technology-enhanced simulation training with no intervention and found large effects for knowledge, skills and behaviours, and moderate effects for patient-related outcomes [2]. The fact that simulation-based training is better than no training might not be a big surprise; to quote Geoff Norman in his editorial *Data dredging, salami-slicing, and other successful strategies to ensure rejection: twelve tips on how to not get your paper published*: ‘We don’t need to compare something with nothing…We’ll accept without proof that some education is better than none’ [3]. However, a recent randomized controlled trial found that approximately 4 h of virtual-reality simulator training was significantly more efficient than a half day of supervised apprenticeship training on patients [4]. It is no longer of question *if* we should practice on simulators but *how* simulation-based training should be implemented.

First of all, it is important to acknowledge that simulation-based training cannot replace traditional apprenticeship training. New surgical trainees must be supervised by more experienced colleagues during their first real procedures just as simulator trained pilots start out as co-pilots. Furthermore, we need to rethink the extreme focus on simulation equipment that has been the hallmark of the literature concerning advanced technical skills training. Nesbitt et al. carefully list the different pros and cons of synthetic models, animal models, virtual reality simulators, and human cadavers and even describe an ideal endovascular training model. Rightfully, they end up concluding that: ‘The biggest current barrier to the routine integration of simulation into endovascular training is the lack of an agreed curriculum’—not the lack of equipment. There is no doubt that the ‘boys and their toys’ phenomenon has resulted in many expensive simulators being covered in dust once the initial enthusiasm has disappeared. A viable training programme based on the best available evidence should be planned before investing time and money in simulation-based training.

Creating professional simulation centres that are used by many departments and hospitals is an efficient way of countering potential problems with costs, licenses, logistics etc [5]. However, we totally agree with Nesbitt et al. that: ‘Simulation should not be a one-off training exercise’. Distributed learning where the training sessions are spaced out over several days is more efficient than massed practice which limits the maximum practical distance between the trainees and the simulation centre [6]. Fortunately, two trainees can share a simulator (dyad training) and a busy (and expensive) consultant does not need to be present at all times [7]. Directed, self-regulated learning where the trainees are allowed to make their own experiences and learn from their mistakes can improve retention compared with instructor-led training [8]. No matter how the training is conducted it is essential to end each programme with a simulation-based test. Not all trainees learn at the same pace and training to a pre-defined criterion is the only way to ensure basic competency before performance on patients. Furthermore, final testing is motivating and improves retention. Mastery learning produces strong and lasting effects and mandatory training and certification programmes are necessary to ensure the maximum gain from simulation-based skills training [9].

Simulation-based tests with solid evidence of validity and defensible pass/fail scores are a prerequisite for mastery learning. The Standards for Educational and Psychological Testing recommend to view validity as a unitary concept and to abandon the historical nomenclature (*face validity, construct validity* etc.) [10]. These recommendations have been around for more than 15 years and it is about time surgeons replace the outdated framework of validity with a contemporary one, such as Messick’s or Kane’s [11]. The design of the test is also important. Evidence suggests that global rating scales are better than checklists in capturing nuanced elements of expertise and that incompetent trainees can achieve high checklist scores despite committing serious procedural errors [12].

In conclusion, there is overwhelming evidence for the efficacy of simulation-based training in clinical skills training. Simulation must be integrated in the training curriculum as distributed training sessions with the possibility of directed, self-regulated learning in professional training facilities. Simulation-based training to proficiency should be mandatory before trainees are allowed to perform procedures on patients.
